# Mesencephalic Electrical Stimulation Reduces Neuroinflammation after Photothrombotic Stroke in Rats by Targeting the Cholinergic Anti-Inflammatory Pathway

**DOI:** 10.3390/ijms22031254

**Published:** 2021-01-27

**Authors:** Michael K. Schuhmann, Lena Papp, Guido Stoll, Robert Blum, Jens Volkmann, Felix Fluri

**Affiliations:** 1 Department of Neurology, University Hospital of Würzburg, 97080 Würzburg, Germany; Schuhmann_M@ukw.de (M.K.S.); Papp_L@ukw.de (L.P.); Stoll_G@ukw.de (G.S.); Volkmann_J@ukw.de (J.V.); 2 Institute of Neurobiology, University Hospital of Würzburg, 97078 Würzburg, Germany; Blum_R@ukw.de

**Keywords:** photothrombotic stroke, deep brain stimulation, mesencephalic locomotor region, neuroinflammation, choline acetyltransferase, alpha-7 nicotinic acetylcholine receptor

## Abstract

Inflammation is crucial in the pathophysiology of stroke and thus a promising therapeutic target. High-frequency stimulation (HFS) of the mesencephalic locomotor region (MLR) reduces perilesional inflammation after photothrombotic stroke (PTS). However, the underlying mechanism is not completely understood. Since distinct neural and immune cells respond to electrical stimulation by releasing acetylcholine, we hypothesize that HFS might trigger the cholinergic anti-inflammatory pathway via activation of the α7 nicotinic acetylcholine receptor (α7nAchR). To test this hypothesis, rats underwent PTS and implantation of a microelectrode into the MLR. Three hours after intervention, either HFS or sham-stimulation of the MLR was applied for 24 h. IFN-γ, TNF-α, and IL-1α were quantified by cytometric bead array. Choline acetyltransferase (ChAT)^+^ CD4^+^-cells and α7nAchR^+^-cells were quantified visually using immunohistochemistry. Phosphorylation of NFĸB, ERK1/2, Akt, and Stat3 was determined by Western blot analyses. IFN-γ, TNF-α, and IL-1α were decreased in the perilesional area of stimulated rats compared to controls. The number of ChAT^+^ CD4^+^-cells increased after MLR-HFS, whereas the amount of α7nAchR^+^-cells was similar in both groups. Phospho-ERK1/2 was reduced significantly in stimulated rats. The present study suggests that MLR-HFS may trigger anti-inflammatory processes within the perilesional area by modulating the cholinergic system, probably via activation of the α7nAchR.

## 1. Introduction

After focal cerebral ischemia, proinflammatory cytokines such as interleukin (IL)-1 or tumor necrosis factor (TNF)-α are strongly induced [1,2]. We and others have shown that invasive electrical stimulation of distinct brain areas such as the fastigial nucleus, cerebral cortex, or mesencephalic locomotor region (MLR) may attenuate neuroinflammation [3,4,5]. In particular, high-frequency stimulation (HFS) within or close to the cuneiform nucleus (Cn) resulted in a significant reduction of proinflammatory cytokines and chemokines in the perilesional area of a photothrombotic stroke (PTS) ipsilesional to the stimulated site [4]. However, the effect of electrical stimulation on putative neural regulation of inflammatory processes is not well understood. In this context, vagus nerve stimulation (VNS) might serve as a paradigm in which electrical stimulation finally results in immunomodulation. There is a growing body of literature reporting that VNS has an effect on peripheral immune activation and proinflammatory cytokines [6,7,8] as well as on the blood-brain barrier [9] and infarct size of experimental stroke [10,11,12]. VNS triggers the release of acetylcholine (ACh) from a subset of CD4^+^ T cells within the spleen [13]. ACh binds to the nicotinic ACh receptor subunit α7 (α7nAchR) expressed on macrophages and reduces the synthesis of proinflammatory cytokines [13]. Stimulation of α7nAchR is also associated with the modulation of different signaling pathways, namely the JAK2/STAT3/NFκB [14,15], JAK2/PI3/Akt [16], and ERK1/2/PKA pathways [17]. With these considerations in mind, we investigated in a rat PTS model whether (i) MLR-HFS ipsilateral to the photochemically induced lesion results in a decrease of proinflammatory cytokines; (ii) the putative reduction of proinflammatory agents is associated with a modulation of the cholinergic system, determined indirectly by the measurement of choline acetyltransferase (ChAT)-positive CD4^+^-cells; and (iii) whether anti-inflammatory processes might be mediated via the α7nAchR signaling by determining key proteins of the NFκB, Akt, Stat3, and ERK1/2 pathway.

## 2. Results

### 2.1. MLR-HFS Does Not Impact Infarct Size but Attenuates Perilesional Proinflammatory Cytokine Concentrations

**Figure 1:**
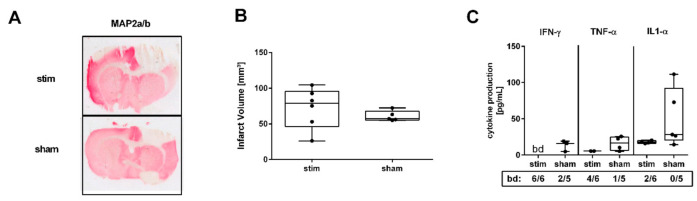
High-frequency stimulation (HFS) of the mesencephalic locomotor region (MLR) 3 h after induction of photothrombotic stroke has no impact on infarct growth but is associated with a significant decrease of proinflammatory factors in the perilesional region 27 h after induction of photothrombosis. (**A**) Representative Map2a/b-immunostained brain sections of a stimulated rat (stim, 40 µA, upper panel) and an unstimulated rat (sham, lower panel). (**B**) Assessment of lesion size. Infarct volumes are similar among stimulated and unstimulated animals (*n* = 5–6/group). (**C**) Absolute amounts of interferon gamma (IFN-γ), tumor necrosis factor alpha (TNF-α), and interleukin (IL)-1α were quantified in 3 mm-thick brain slices of the perilesional region within the sensorimotor cortex 1 mm before bregma. Stimulated rats showed lower levels of IFN-γ, TNF-α, and IL-1α compared to unstimulated rats (*n* = 5–6/group). bd—beyond detection limit.

**Figure 2:**
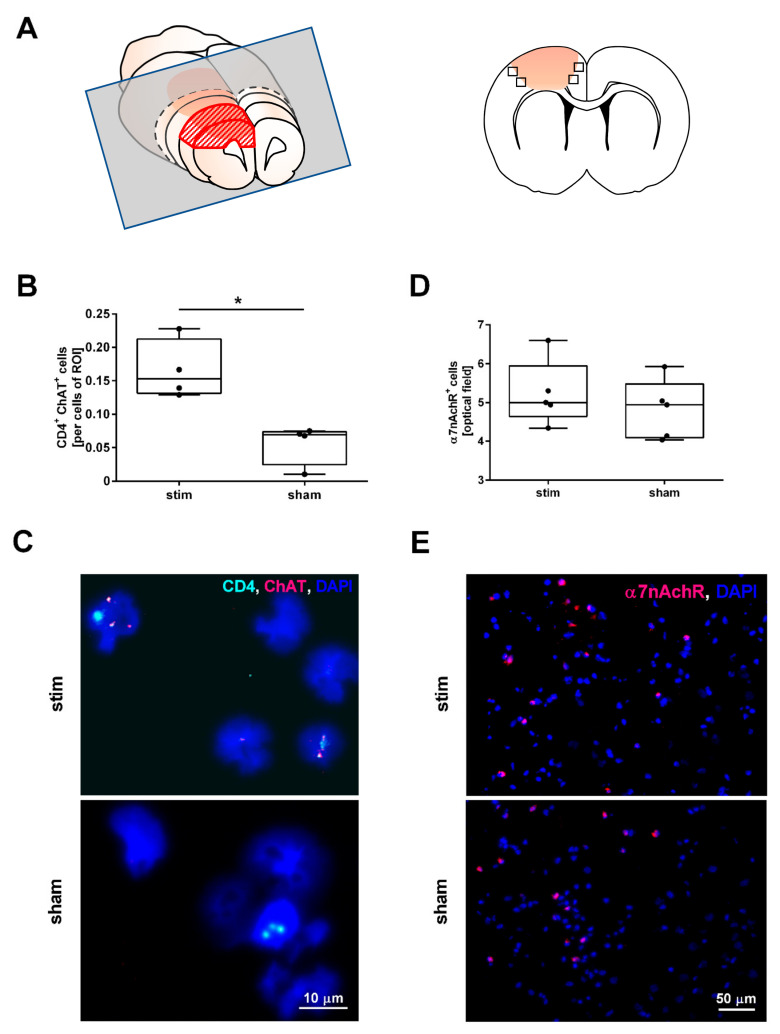
High-frequency stimulation of the mesencephalic locomotor region changes the number of cells expressing choline acetyltransferase (ChAT) in the perilesional region during the acute phase of photothrombotic stroke but does not change the number of cells expressing alpha nicotinic acetylcholine receptors (α7nAchR). (**A**) Scheme of a 3 mm-thick coronal rat brain slice cut 1 mm before Bregma (grey rectangular) used for cytospin analyses (**left panel**); brain section indicating the recorded fields of vision (squares) used for the fluorescence immunostaining (**right panel**). (**B**) Quantification of ChAT^+^/CD4^+^-cells in cytospin specimens originating from brain tissue surrounding the photothrombotic lesion. MLR-HFS significantly increased the number of ChAT^+^/CD4^+^-cells in the border zone of the PTS compared to sham-stimulation; Mann–Whitney test; * *p* < 0.05 (*n* = 4/group). (**C**) Visualization of cells expressing CD4 (cyan) and ChAT (magenta) within a cytospin specimen originating from the perilesional area of stimulated (**upper panel**) and unstimulated (**lower panel**) animals using fluorescence in situ hybridization. (**D**) Quantification of α7nAchR^+^-cells in the perilesional area yielded similar results in the two treatment groups; Mann–Whitney test; *p* = n.s. (*n* = 5/group). (**E**) Visualization of α7nAchR (magenta) expressing cells of stimulated (**upper panel**) and unstimulated (**lower panel**) animals in the perilesional area.

Since MLR-HFS was started in the acute phase of ischemia, i.e., 3 h after the induction of PTS, HFS may have an influence on infarct growth. Therefore, 24 h after beginning MLR-HFS, the lesion size of stimulated and unstimulated rats was determined on Map2a/b-stained brain slices using planimetry (Figure 1A). Lesion size did not differ significantly between the two groups (stim vs. sham: 78.99 [interquartile range (IQR) 46.33–95.60] vs. 57.24 [IQR 55.46–67.71] mm^3^, *p* = n.s., *n* = 5–6/group, Figure 1B). Cerebral ischemia activates innate immune receptors on microglia/macrophages and other cells resulting in the release of cytokines and chemokines such as IFN-γ, TNF-α, and IL-1α [18]. These proinflammatory agents modulate tissue damage early after induction of experimental stroke [18,19]. As a proof of concept, we investigated whether MLR-HFS starting in the acute phase of ischemia and continued for 24 h was associated with an attenuation of proinflammatory mediators, namely IFN-γ, TNF-α, and IL-1α in the cerebral tissue surrounding the photothrombotic lesion. A tissue sample of each cerebral hemisphere (2 mm-thick sections) was harvested 1 mm anterior to bregma (Figure 2A, left). IFN-γ, TNF-α, and IL-1α were quantified using a fluorescent bead immunoassay. MLR-HFS for 24 h markedly reduced levels of proinflammatory cytokines (IFN-γ stim vs. sham: bd vs. 16.15 [IQR 4.84–19.19] pg/mL, TNF-α stim vs. sham: 5.62 [IQR 5.62–5.62] vs. 16.52 [IQR 6.62–24.91] pg/mL, IL-1α stim vs. sham: 17.83 [IQR 16.33–19.98] vs. 28.37 [IQR 20.50–92.28] pg/mL, *n* = 5–6/group; Figure 1C).

### 2.2. MLR-HFS Is Associated with an Increased Number of ChAT Expressing Cells but Does Not Change the Amount of α7nAchR-Positive Cells

In the next set of experiments, we examined the impact of MLR-HFS on the synthesis of ACh by assessing the ChAT expression of CD4^+^-cells residing in the perilesional area of the PTS. For this purpose, we generated cytospins of brain tissue dissected 1–4 mm anterior from bregma (Figure 2A). In stimulated rats, ChAT^+^CD4^+^-cells were significantly more abundant in the lesioned brain hemisphere compared to sham animals (ChAT^+^CD4^+^-cells stim vs. sham: 0.15 [IQR 0.13–0.21] vs. 0.07 [IQR 0.02–0.07], ChAT^+^CD4^+^-cells/cells of ROI, * *p* < 0.05, *n* = 4/group; Figure 2B,C). The receptor polypeptide α7nAchR, responding to the neurotransmitter acetylcholine, is expressed in many regions of the central and peripheral nervous system but also on non-neuronal cells including astrocytes and microglia [20,21,22]. Activation of α7nAchR via receptor agonists results in anti-inflammatory effects such as a decrease in the synthesis of cytokines and chemokines [23,24]. Therefore, we examined whether MLR-HFS impacts the amount of α7nAchR expressing cells. However, quantification of α7nAchR^+^-cells did not yield a difference between stimulated and unstimulated rats (α7nAchR^+^-cells stim vs. sham: 5.00 [IQR 4.64–5.95] vs. 4.94 [4.09–5.48], α7nAchR^+^-cells/optical field, *p* = n.s., *n* = 5/group, Figure 2D,E).

### 2.3. MLR-HFS Modulates ERK1/2 Signaling Pathway

**Figure 3:**
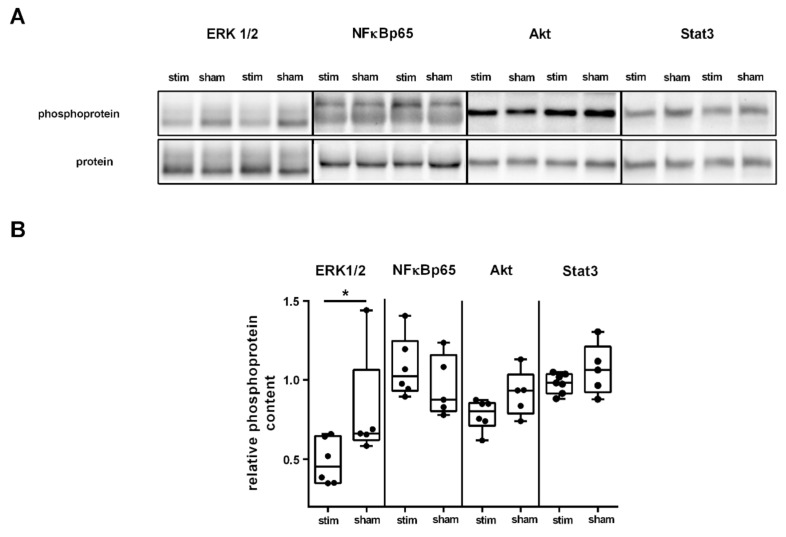
High-frequency stimulation of the mesencephalic locomotor region modulates distinct signaling pathways in the acute phase after photothrombotic stroke. (**A**) Representative Western blot of ERK1/2, NFĸB, Akt, and Stat3 originating from perilesional tissue of the ipsilesional cortex and densitometric quantification of the relative amount of phosphoprotein among the two groups. (**B**) Quantification of four key proteins involved in pathways modulating neuroinflammation within the perilesional area of the sensorimotor cortex. Western blot analysis yielded a significantly lower level of phosphorylated ERK1/2 in animals undergoing MLR-HFS compared to unstimulated rats (*n* = 5–6/group), which indicates a modulating effect of MLR-HFS on this pathway; the amount of phosphorylated Akt, NFĸB, and Stat3, however, did not differ significantly between these two groups. * < 0.05.

α7nAchR has been shown to modulate intracellular signaling pathways, including the JAK2/STAT3/NFκB [25], JAK2/PI3/Akt [26], and ERK1/2/PKA pathways [17] and thus leads to anti-apoptotic, antioxidative, and anti-inflammatory effects. Triggered by the finding that MLR-HFS is associated with a reduction of IFN-γ, TNF-α, and IL-1α, we investigated whether key proteins of the aforementioned immunomodulatory pathways are modulated by MLR-HFS compared to sham-stimulation. MLR-HFS significantly reduced ERK1/2 phosphorylation 27 h after induction of PTS (ERK1/2 stim vs. sham: 0.45 [IQR 0.35–0.66] vs. 0.66 [IQR 0.62–1.07] ratio (pERK1/2)/(ERK1/2), * *p* < 0.05, *n* = 5–6/group), whereas the relative phosphoprotein content of NFκBp65, Akt, and Stat3 was similar among both, the stimulated and unstimulated group (NFκBp65 stim vs. sham: 1.02 [IQR 0.93–1.25] vs. 0.88 [IQR 0.80–1.16] ratio (pNFκBp65/NFκBp65), *p* = n.s., Akt stim vs. sham: 0.80 [IQR 0.71–0.86] vs. 0.93 [IQR 0.79–1.03] ratio (pAkt/Akt), *p* = n.s., Stat3 stim vs. sham: 0.98 [IQR 0.92–1.04] vs. 1.06 [IQR 0.92–1.21] ratio (pStat3/Stat3), *p* = n.s., *n* = 5–7/group; Figure 3A,B).

## 3. Discussion

Here, we show that continuous MLR-HFS, beginning 3 h after onset of cortical PTS and continued for 24 h, alleviates neuroinflammation in rats by affecting the cholinergic anti-inflammatory pathway. One main finding of this study is a decrease in the amounts of IFN-γ, TNF-α, and IL-1α within the perilesional area of stimulated rats compared to controls, indicating that MLR-HFS even has a remote effect on neuroinflammation. These data are also in line with the results of our recently published study demonstrating that MLR-HFS after PTS significantly reduces IL-17 and IL-18 as well as monocyte chemotactic protein-1 (MCP-1) and chemokine ligand 1 (CXCL-1) in the perilesional area of stimulated rats [4]. Interestingly, invasive electrical stimulation of other brain regions also attenuates neuroinflammation: high-frequency deep brain stimulation (DBS) (130 Hz) of the anterior thalamic nucleus resulted in a decrease of TNF-α and IL-1β in a rat model of epilepsy [21]. Similarly, DBS of the subthalamic nucleus revealed a reduced level of IL-1β in the substantia nigra of a rat model of Parkinson’s disease [20].

The mechanism by which DBS modulates neuroinflammation in the central nervous system remains elusive. However, there is growing knowledge of how electrical stimulation of the peripheral nervous system interferes with inflammatory processes. When the vagus nerve is stimulated, impulses travel along vagal efferents to the celiac ganglia and finally to the splenic nerve. Its terminals release norepinephrine, which triggers the synthesis of ACh from a subset of CD4^+^ T cells expressing ChAT. Similar to the effect of VNS, we found a significant increase of ChAT^+^ CD4^+^ T cells after MLR-HFS as well as a reduction of proinflammatory cytokines, which may support the hypothesis of a cerebral cholinergic anti-inflammatory pathway. Recent studies also point toward a cholinergic anti-inflammatory pathway in the brain: ACh and nicotine pretreatment inhibited lipopolysaccharide-induced release of TNF-α from cultured murine microglia [22]. This hypothesis is also supported by animal experiments, showing that acetylcholine esterase (AChE) inhibitors dampen glial activation and synthesis of proinflammatory cytokines in a rodent model of experimental autoimmune encephalomyelitis [23] as well as in a rat model of transient focal cerebral ischemia [24].

Several studies suggest that α7nAChRs are involved in the regulation of cytokine production, showing that ACh interacts with α7nAChRs on macrophages and suppresses the synthesis of proinflammatory cytokines and inflammation [6,8,13]. In the brain, α7nAChRs are found in both neurons [27] and non-neuronal cells such as microglia [28], astrocytes [29], and endothelial cells [30]. When α7nAChRs are stimulated by their agonist choline in a rat stroke model of permanent middle cerebral artery occlusion (MCAO), an elevated amount of α7nAChRs was recognized as well as a reduction of infarct volume and neurological deficits [31]. In the present study, the number of α7nAChR-expressing cells in the perilesional area did not differ significantly between stimulated and unstimulated animals, indicating that MLR-HFS does not regulate the expression of α7nAChR. 

As a further result, we found a significant increase of ChAT^+^CD4^+^ T cells. Interestingly, a recently published study by Huang and coworkers [32] revealed similar findings: When high-frequency DBS (100 Hz) was applied in the nucleus basalis of Meynert of a transgenic mouse model of Alzheimer’s disease, the activity of ChAT increased whereas that of AChE decreased after high-frequency DBS. Hence, the effect of MLR-HFS on the cerebral cholinergic pathway might be explained by a higher expression of ChAT and, therefore, by a higher concentration of ACh. 

An increased level of ACh might highly saturate α7nAChRs and, thus, strengthen the efficacy of these receptors on intracellular signaling pathways. In particular, activation of the JAK2/STAT3 signaling pathway may contribute to neuronal damage following transient cerebral ischemia in rats [33]. The NFκB signaling pathway regulates the synthesis of proinflammatory mediators at the level of gene transcription after MCAO [34]. Recent studies suggest that α7nAChR-signaling exerts an anti-inflammatory effect via the JAK2/STAT3 and NFκB pathway [14,15]. The JAK2/PI3/Akt pathway, which is also modulated by α7nAChRs, is associated with anti-inflammatory and antioxidative cellular strategies [26,35]. MLR-HFS beginning in the acute phase of PTS and continued for 24 h did not reveal a change in phosphorylation of NFκB. Of note, STAT3 activation primarily occurred during the reperfusion phase rather than during the ischemic phase of stroke [36]. Since our measurements were not performed in brain tissue harvested during the reperfusion phase, activation of STAT3 is therefore not likely to be detected, especially in the subacute phase of ischemia. Additionally, NFκBp65 is significantly induced at 6 h after permanent MCAO and continuously increases with a peak at 24 h, and thereafter decreases [37]. Similarly, phospho-Akt was markedly elevated in a transient MCAO model at 4 h of reperfusion and decreased by 24 h. The spontaneous decrease of these molecules already 24 h after onset of stroke and, thus, before finishing MLR-HFS (27 h after surgery), might partly explain the lacking difference between stimulated and unstimulated animals. 

Furthermore, α7nAChRs are involved in the ERK1/2/PKA signaling pathway, which has been identified as a regulator of cellular survival [17]. Inhibition of ERK 1/2 activation reduces infarct size and neurological deficits after focal ischemia in rodents, indicating that the ERK 1/2 pathway contributes to ischemic cerebral injury [38]. After applying MLR-HFS for 24 h, phospho-ERK 1/2 was significantly reduced in the perilesional area compared to unstimulated controls and may explain the low expression of proinflammatory cytokines after MLR-HFS in the acute phase of PTS, as demonstrated in the present study. These findings may also corroborate the results of another study reporting that electrical high-frequency stimulation of the nucleus basalis of Meynert inhibits the ERK1/2 signaling pathway in a mouse model of Alzheimer’s disease [32]. The authors of this study were able to demonstrate that the ERK1/2 inhibitor U0126, administered to mice after DBS, weakened the neuroprotective effects of HFS [32]. This observation underlines that HFS may interfere with the regulation of the ERK1/2 signaling pathway and thus exerts neuroprotective effects.

Our findings may further raise the question of how MLR-HFS exerts an effect even in cerebral regions remote from the stimulating site. Importantly, the MLR provides no direct axonal projections to the sensorimotor cortex [39,40]. Nevertheless, the MLR is indirectly connected to the cortex via a relay in the thalamus [39,40]. Furthermore, the PPTg projects cholinergic fibers to the basal forebrain [39,41], which is also the origin of cholinergic projections to the sensorimotor cortex [42,43]. However, whether MLR-HFS elicits production of ACh following an increase of ChAT^+^-cells remains speculative.

This study has several limitations. First, only HFS (130 Hz) was used to investigate a putative effect of DBS on the cholinergic anti-inflammatory pathway. However, a recently published study revealed that especially HFS (100 Hz) of the nucleus basalis of Meynert resulted in significant anti-inflammatory and neuroprotective effects, whereas low frequencies did not. Further studies are yet required to examine whether low frequencies (e.g., 10–50 Hz) have a similar effect on neuroinflammation compared to HFS. Second, only a small number of animals were used for each experiment, which might affect the result of the different experiments. Third, we investigated only the effect of MLR-HFS when started 3 h after onset of PTS and continued for up to 24 h. The effect of MLR-HFS starting at earlier or later times after intervention as well as different durations of MLR-HFS should be examined in future studies.

## 4. Materials and Methods

### 4.1. Animals

Ten- to 12-week-old male Wistar rats (*n* = 29; Charles River, Sulzfeld, Germany), were used in this study. Before intervention, rats were housed under a 12 hour light/dark cycle (light on 06:00–18:00) with an ambient temperature of 22 ± 0.5 °C for seven days. Rats were provided with food and water ad libitum. All animal experiments were approved by the institutional review board of the Julius-Maximilians-University Würzburg and by the local authorities at the Regierung von Unterfranken, Würzburg, Germany (TVA55.2-2531.01-102/13; approved on 2 October 2013). The study was performed according to the ARRIVE guidelines (Animal Research: Reporting of In Vivo Experiments; https://www.nc3rs.org.uk/arrive-guidelines). Before starting surgery, animals were randomly divided into a sham-stimulated, i.e., control group (*n* = 14) and in a group that underwent MLR-HFS (*n* = 15).

### 4.2. Induction of Photothrombotic Stroke

A photothrombotic lesion was inflicted within the right cerebral cortex of all animals as described recently [44]. Briefly, after induction of anesthesia (isoflurane 2.5%), rats were fixed in a stereotactic frame and the skull was exposed. Aluminum foil with an aperture was put on the head using the coordinates [45] encompassing the right sensorimotor cortex (5 mm anterior to 5 mm posterior and 0.5 to 5.5 mm lateral to the Bregma). Rose Bengal (Sigma-Aldrich, St. Louis, MO, USA) dissolved in 0.9% NaCl was injected intravenously. Thereafter, the sensorimotor cortex was illuminated for 15 min with a cold light source (Olympus KL1500LCD, Mainz, Germany) which was positioned over the aluminum foil. Body temperature was maintained at 37 ± 0.5 °C by a feedback-controlled heating system during the whole surgery.

### 4.3. Microelectrode Implantation

After inducing a photothrombotic stroke, a stimulating monopolar microelectrode was inserted in the right MLR (coordinates: 7.8 mm posterior, 2.0 mm lateral, and 5.8 mm ventral to the bregma) as described elsewhere [44]. Briefly, a microelectrode (FHC Inc., Bowdoin, ME, USA) was implanted into the MLR using a micromanipulator. Then, the electrode was attached to the skull with anchor screws and dental cement. A plug (GT-Labortechnik, Arnstein, Germany) was put on the freely ending pin of the electrode and fixed with additional dental cement. After wound closure, animals were put in a warmed cage and allowed to wake up. Histopathological analysis verified electrode tip placement within or very close to the Cn.

### 4.4. High-Frequency Stimulation of the Mesencephalic Locomotor Region

Three hours after onset of cortical photothrombosis, MLR-HFS was started in the group of stimulated rats for 24 h using a stimulus generator (STG 4002, Multichannel Systems, Reutlingen, Germany). The following stimulus parameters were applied: frequency, 130 Hz; pulse length, 60 µs; pulse shape: monophasic square waves. The threshold current amplitude for each rat was assessed according to the locomotor behavior under MLR-HFS. The stimulating amplitude was determined, as follows: beginning with 20 µA, the intensity was increased in steps of 10 µA until maximal locomotion was observed. The lowest amplitude evoking locomotor behavior was chosen for the 24 h MLR-HFS (for details see [44]). At the same time, control animals were connected with a stimulus generator, which was turned off. No differences in behavior were observed between electrically stimulated rats and controls during the whole experiment.

### 4.5. Collection of Cerebral Tissue

After finishing the 24 h MLR-HFS (i.e., 27 h after photochemically induced stroke), animals were anesthetized by injecting pentobarbital and perfused transcardially with phosphate-buffered saline (PBS) for 4 min. Thereafter, brains were removed. One millimeter anterior to bregma, a 3 mm-thick brain slice was cut from the brain. The cerebral cortex comprising the perilesional area was dissected from the subcortical tissue for further analysis.

### 4.6. Brain Cell Separation

Brain tissue was dissociated mechanically, and cells were isolated from the interface of a 30–50% Percoll (Amersham Biosciences, Buckinghamshire, UK) density gradient as described elsewhere [46], followed by a cytospin slide preparation.

### 4.7. Infarct Quantification

Infarct size was quantified as follows: cryo-embedded brain tissue 5 mm anterior and posterior to the bregma were cut into 10 µm-thick brain slices using a microtome (Leica SM 2000R, Wetzlar, Germany). Every tenth brain slice was stained and digitalized using a scanner (Epson Perfection V500 Photo, Epson, Germany). Brain hemispheres and the area of the lesion were traced manually on each scan using ImageJ Analysis Software v1.52e (National Institutes of Health, Bethesda, MD, USA, https://imagej.nih.gov/ij/). The areas were then summed and multiplied by the slice-slice interval.

### 4.8. Immunohistochemistry

Immunohistochemistry of cryo-embedded brain slices (10 µm thick) was performed as described elsewhere in detail [47]. In order to visualize alpha7 nicotinic acetylcholine receptor (α7nAchR), brain slices were incubated using anti-rat-nAChR α7 (CHRNA7; Alomone Labs, Jerusalem/Israel, catalogue #ANC-007), and nuclei of cells were stained using 4,6-diamidino-2-phenylindole (DAPI) (Sigma-Aldrich, St Louis, MO, USA, catalogue #D9542). Quantification of α7nAchR-positive cells was carried out on 5 subsequent slices per animal with an interval of 100 µm by counting 4 optical fields per cortex region. Sections were analyzed under a microscope (Nikon Eclipse 50i, Nikon Instruments Europe BV, Amsterdam, Netherlands) equipped with a charge-coupled device camera using 20-fold magnification.

### 4.9. Fluorescence in Situ Hybridization (FISH)

To identify choline acetyltransferase (ChAT)-positive immune cells in cytospin preparations of brain tissue, we performed fluorescence in situ hybridization (FISH) using the RNAscope Multiplex Fluorescent v2 Assay according to the manufacturer’s instructions (Advanced Cell Diagnostics, Milan, Italy; catalogue #323100). Target probes for ChAT (RNAscope probe Rn-Chat-C2, catalogue #430111-C2) were designed by Advanced Cell Diagnostics. After amplification and label application, sections were counterstained with 4,6-diamidino-2-phenylindole (Sigma-Aldrich, St Louis, MO, USA; catalogue #D9542). Images were acquired with a Leica DMi8 microscope (Leica Microsystems, Wetzlar, Germany) (magnification = 40×).

### 4.10. Western Blot Assays

Western blot analyses were performed according to standard procedures using antibodies against phospho-ERK1/2 (Cell Signaling, Danvers, MA, USA, catalogue #4370), ERK1/2 (Cell Signaling, Danvers, MA, USA, catalogue #4695), phospho-NFĸBp65 (Cell Signaling, Danvers, MA, USA, catalogue #3039), NFĸBp65 (Cell Signaling, Danvers, MA, USA, catalogue #8242), phospho-Akt (Cell Signaling, Danvers, MA, USA, catalogue #4051), Akt (Cell Signaling, Danvers, MA, USA, catalogue #9272), phospho-STAT3 (Cell Signaling, Danvers, MA, USA, catalogue #9145), and STAT3 (Cell Signaling, Danvers, MA, USA, catalogue #9139) as described elsewhere [48]. Brain tissue used for Western blot analyses originated from the perilesional region.

### 4.11. Cytokine Quantification

We quantified the production of interferon gamma (IFN-γ), tumor necrosis factor alpha (TNF-α), and interleukin (IL)-1α in rat brain lysates originating exclusively from the perilesional region while using a fluorescent bead immunoassay (LEGENDplex, Biolegend, San Diego, CA, USA), according to the manufacturer’s instructions.

### 4.12. Statistical Analysis

For statistical analysis, the GraphPad Prism v6.0 software package (GraphPad Software) was used. Results are given as median and the interquartile range (IQR) is indicated for each value. Data were analyzed by the Mann–Whitney test for nonparametric data. *p* < 0.05 was considered statistically significant.

## 5. Conclusions

In the acute phase of PTS, MLR-HFS was associated with a significant reduction of proinflammatory cytokines in the region surrounding the PTS. Since an increased number of CD4^+^ T cells expressing ChAT (i.e., a surrogate marker of ACh) was detected in the perilesional area of stimulated rats, we suggest that the underlying mechanism of MLR-HFS-triggered immunomodulation is the cholinergic anti-inflammatory pathway in the brain. Within this signaling cascade, stimulated α7nAchRs orchestrates different subcellular pathways, in particular, the Erk1/2 pathway. Continuous MLR-HFS for 24 h resulted in a significant decrease of phospho-Erk1/2 and thus, contributes to the anti-inflammatory processes within the area surrounding the PTS. Hence, MLR-HFS not only trigger locomotor behavior but might also be involved in the modulation of subcellular pathways.

## Data Availability

The analyzed data sets generated during the study are available from the corresponding author on reasonable request.

## Abbreviations

α7nAchR	alpha 7 nicotinic acetylcholine receptor
ACh	acetylcholine
AChE	acetylcholine esterase
ChAT	choline acetyltransferase
DBS	deep brain stimulation
ERK1/2	extracellular signal-regulated kinase 1/2
HFS	high-frequency stimulation
IL	interleukin
INF-γ	interferon gamma
MLR	mesencephalic locomotor region
NFκB	nuclear factor kappa-light-chain-enhancer of activated B cells
PTS	photothrombotic stroke
TNF-α	tumor necrosis factor alpha
VNS	vagus nerve stimulation
